# Procalcitonin kinetics in Gram-negative bloodstream infections and its value for infection management

**DOI:** 10.1099/jmm.0.002168

**Published:** 2026-05-11

**Authors:** Saied Ali, Niamh Mullane, Nicki Rees, David Greville, Meadhbh Collison, Patrick Twomey, Silke Ryan, Kirsten Schaffer

**Affiliations:** 1 Department of Clinical Microbiology, St Vincent’s University Hospital, Dublin, Ireland; 2 Department of Microbiology, Royal College of Surgeons in Ireland, Dublin, Ireland; 3 Department of Clinical Microbiology, Mercy University Hospital, Cork, Ireland; 4 Department of Clinical Microbiology, National Maternity Hospital, Dublin, Ireland; 5 Department of Clinical Chemistry, St Vincent’s University Hospital, Dublin, Ireland; 6 Department of Respiratory Medicine, St Vincent’s University Hospital, Dublin, Ireland

**Keywords:** antimicrobial therapy, bloodstream infection, C-reactive protein, *Enterobacterales*, procalcitonin, sepsis

## Abstract

**Introduction.** Gram-negative bloodstream infections (BSIs), particularly those caused by *Enterobacterales*, are associated with significant morbidity and mortality. Procalcitonin (PCT) has been proposed as a biomarker to guide antimicrobial management, although its role in BSIs remains unclear.

**Hypothesis/Gap Statement.** While PCT has been widely studied in respiratory infections and critical illness, the clinical utility of early PCT kinetics in guiding antimicrobial adequacy and source control in BSIs is not well defined.

**Aim.** To evaluate whether early changes in PCT are associated with antimicrobial appropriateness, source control and clinical outcomes in *Enterobacterales* BSIs and to compare these findings with C-reactive protein (CRP).

**Methodology.** We conducted a cohort study of 170 hospitalized adults with monomicrobial *Enterobacterales* BSIs. Serum PCT and CRP were measured at blood culture positivity (day 0) and serially on days 2 and 5. Biomarker kinetics were analysed using non-parametric methods, including proportional change from baseline to day 5. Associations with clinical variables and outcomes were assessed, and multivariable logistic regression was used to evaluate relationships between baseline biomarker concentrations and illness severity.

**Results.** Median baseline PCT was 5.81 ng ml^−1^ (IQR 1.28–38.19) and CRP 124.1 mg l^−1^ (IQR 60.5–203.0). Both biomarkers increased from day 0 to day 2 and declined by day 5 (*P*<0.001). Absolute PCT concentrations were lower in patients receiving inappropriate empiric therapy (*P*=0.016), but neither proportional nor absolute changes in PCT or CRP were associated with antimicrobial appropriateness, source control, infecting organism or infection source. Baseline PCT was independently associated with intensive care unit admission (adjusted odds ratio, 1.24; 95% confidence interval, 1.03–1.49) and a quick Sequential Organ Failure Assessment score of two or greater (adjusted odds ratio, 1.21; 95% confidence interval, 1.00–1.45), whereas CRP was not. Neither PCT nor CRP kinetics were associated with 30-day mortality (*P*=0.677 and *P*=0.475, respectively).

**Conclusion.** Early PCT kinetics do not provide clinically useful information to guide antimicrobial management in *Enterobacterales* BSIs. While baseline PCT reflects illness severity, serial measurements appear to reflect host inflammatory response rather than treatment effect, limiting their utility in early clinical decision-making.

## Introduction

Gram-negative bloodstream infections (BSIs) are a major cause of morbidity and mortality worldwide [1]. *Enterobacterales* account for approximately one-quarter to one-half of cases and remain the leading cause of healthcare-associated bacteraemia [2,3]. The increasing emergence and dissemination of extended-spectrum *β*-lactamases and carbapenemases within *Enterobacterales* have further complicated management, with antimicrobial resistance posing a substantial burden across both community- and hospital-acquired infections [4,5]. As a result, timely optimization of antimicrobial therapy and effective source control are critical determinants of outcomes [6,7].

Procalcitonin (PCT), a precursor of calcitonin, is released in response to bacterial infection and demonstrates greater specificity for bacterial inflammation than C-reactive protein (CRP) [8,10]. It has been widely evaluated as both a diagnostic adjunct in sepsis and a tool to guide antimicrobial stewardship, particularly in decisions regarding antimicrobial discontinuation [11]. Evidence from randomized and observational studies, largely in respiratory infections and critically ill patients, suggests that PCT-guided strategies reduce antimicrobial exposure without adverse clinical outcomes, with some studies reporting improved survival [11,14]. Its role in BSIs, however, remains less well defined.

In BSIs, PCT concentrations are typically higher in Gram-negative compared with Gram-positive or fungal infections, and increasing levels have been associated with worse outcomes [15,16]. While low PCT concentrations may assist in excluding bacteraemia at thresholds such as 0.5 ng ml^−1^, no single cut-off provides adequate sensitivity across clinical settings [17,18]. Interpretation is further limited by inter-individual variability in host responses, with similar infectious foci producing markedly different PCT levels [9,19, 20].

Attention has therefore shifted towards PCT kinetics, with serial measurements potentially offering greater clinical utility than isolated values in assessing treatment response. This is particularly relevant where early assessment of antimicrobial adequacy and source control is uncertain. CRP remains widely used to monitor response to infection, although its slower kinetics and lower specificity may limit its usefulness in early decision-making [21].

Against this background, the potential role of early biomarker kinetics in informing treatment response remains uncertain. We, therefore, aimed to evaluate whether serial PCT measurements are informative in the early management of Gram-negative BSIs, specifically whether a declining PCT trajectory is associated with appropriate antimicrobial therapy and/or adequate source control and how these trends compare with CRP.

## Methods

### Study setting and patient selection

This study was conducted in a 600-bed tertiary adult referral centre providing acute, chronic and emergency care across a broad range of medical and surgical specialties, including national referral services in cystic fibrosis, oncology and solid organ transplantation. Consecutive hospitalized patients with Gram-negative BSIs were identified through the microbiology laboratory at the time of blood culture positivity. Patients were included where *Enterobacterales* were isolated from blood culture. Only monomicrobial episodes were included.

### Microbiological methods

Blood cultures were processed using the BD BACTEC^™^ FX Blood Culture System [22]. Positive cultures were sub-cultured directly onto solid media. Organisms were identified using matrix-assisted laser desorption ionisation time-of-flight (MALDI-TOF) MS [23]. Antimicrobial susceptibility testing (AST) was performed using automated broth microdilution on the VITEK^®^ 2 (bioMérieux) platform [24]. Direct susceptibility testing from positive blood cultures provided provisional resistance phenotypes for a limited number of agents, with final susceptibility results available following the standard testing. AST results were interpreted in accordance with the most recent European Committee on Antimicrobial Susceptibility Testing criteria [25].

All positive blood cultures are communicated in real time by the clinical microbiology team to the treating clinician at the time of positivity. Antimicrobial advice was provided based on Gram stain results, prior microbiology where available, and the clinical context.

### Data collection and definitions

Data were collected from January 2021 to December 2025. PCT levels were retrospectively measured on serum samples corresponding to the date of blood culture positivity (day 0), with serial measurements obtained prospectively on days 2, 5, 7 and 10 where available. Corresponding CRP values were recorded at the same time points.

Patient demographics were collected, including admission source to classify infections as community- or hospital-acquired. Microbiological data included the organism isolated from blood culture and the presumed source of BSI.

Antimicrobial therapy was recorded, including empiric and subsequent targeted treatment. Empiric therapy was considered appropriate if at least one administered agent demonstrated *in vitro* susceptibility against the BSI isolate on AST.

Source control was assessed over the 10-day follow-up period. Inadequate source control was defined as the presence of an unresolved focus of infection, including undrained collections or unrelieved obstruction, given its potential impact on biomarker kinetics despite appropriate antimicrobial therapy.

Severity of illness was assessed using the quick Sequential Organ Failure Assessment (qSOFA) score at day 0. Clinical outcomes included discharge, admission to intensive care, hospital readmission within 30 days of discharge and 30-day mortality.

### PCT assay

Serum PCT concentrations were measured using an electrochemiluminescence immunoassay for quantitative detection [26]. The assay had a measuring range of 0.02–100 ng ml^−1^, with a limit of quantification of 0.06 ng ml^−1^. Values exceeding the upper limit of the assay were reported by the laboratory as >100 ng ml^−1^ and were assigned a value of 100 ng ml^−1^ for the purpose of analysis, acknowledging the upper limit of quantification.

### CRP assay

Serum CRP concentrations were measured using a particle-enhanced immunoturbidimetric assay [27]. The assay had a measuring range of 0.6–350 mg l^−1^, with a lower limit of quantification of 0.6 mg l^−1^. Values exceeding the upper limit of the assay were reported as >350 mg l^−1^ and were assigned a value of 350 mg l^−1^ for the purpose of analysis, acknowledging the upper limit of quantification.

### Statistical analysis

Data were analysed using SPSS software (version 27, IBM). Biomarker distributions were markedly right-skewed, so continuous variables were summarized as median and interquartile range (IQR) and compared using non-parametric methods. Baseline and follow-up PCT and CRP values were compared between groups using the Mann–Whitney *U* test for two-group comparisons and the Kruskal–Wallis test for comparisons across more than two groups.

Within-patient change in PCT and CRP between day 0 and subsequent time points was assessed using the Wilcoxon signed-rank test. In addition to absolute biomarker values, proportional change from baseline to day 5 was calculated for both PCT and CRP. Day 5 was used for the principal kinetic comparisons because it provided the best balance between early treatment response and biomarker availability.

Exploratory subgroup analyses were performed according to organism and source of BSI. Because *Escherichia coli* accounted for the majority of episodes, organisms were analysed as *E. coli* versus non-*E. coli*. The source was grouped as urinary, intra-abdominal and others.

Multivariable logistic regression was used to assess whether baseline PCT and CRP were independently associated with intensive care unit (ICU) admission, 30-day mortality and qSOFA ≥2, adjusting for age and sex. Because of skewed distributions, baseline biomarker values were log-transformed before entry into regression models.

## Results

### Patient characteristics

A total of 170 patients with monomicrobial Gram-negative BSIs were included. Median age was 72 years (IQR 63 to 81), and 98/170 (57.6%) were male. Ninety-four episodes (55.3%) were community-acquired, and 76/170 (44.7%) were nosocomial.

*E. coli* was the most common BSI isolate, accounting for 107/170 (62.9%) cases, followed by *Klebsiella pneumoniae* in 25/170 (14.7%), *Enterobacter cloacae* in 10/170 (5.9%) and *Proteus mirabilis* in 9/170 (5.3%). The urinary tract was the most common source of BSI, identified in 80/170 (47.1%) episodes, followed by intra-abdominal infection in 63/170 (37.1%) and respiratory infection in 14/170 (8.2%).

Inappropriate empiric antimicrobial therapy at the time of blood culture positivity was present in 18/170 (10.6%) patients, and 43/170 (25.3%) had a documented source control concern. ICU admission occurred in 41/170 (24.1%) patients, and 21/170 (12.4%) died within 30 days.

Clinical, microbiological and outcome characteristics are summarized in Table 1.

**Table 1.** Clinical, microbiological and outcome characteristics of included patients with Gram-negative bloodstream infections

Variable	Overall cohort (*n*=170)
**Age, median (IQR), years**	72 (63–81)
**Male sex, *n* (%)**	98 (57.6)
**Admission source, *n* (%)**	
Community-acquired	94 (55.3)
Nosocomial	76 (44.7)
**Blood culture isolate, *n* (%)**	
*Escherichia coli*	107 (62.9)
*Klebsiella pneumoniae*	25 (14.7)
*Enterobacter cloacae*	10 (5.9)
*Proteus mirabilis*	9 (5.3)
Other *Enterobacterales*	19 (11.2)
**Source of BSI, *n* (%)**	
Urinary tract	80 (47.1)
Intra-abdominal	63 (37.1)
Respiratory	14 (8.2)
Others	13 (7.6)
**Inappropriate empiric therapy, *n* (%)**	18 (10.6)
**Source control concern, *n* (%)**	43 (25.3)
**ICU admission, *n* (%)**	41 (24.1)
**30-day mortality, *n* (%)**	21 (12.4)

### Biomarker availability and overall kinetics

PCT measurements were available in all 170 patients at day 0 and day 2, in 132 patients at day 5, 97 at day 7 and 72 at day 10. CRP measurements were available in 169 patients at day 0, 168 at day 2, 146 at day 5, 117 at day 7, and 93 at day 10.

At baseline, median PCT was 5.81 ng ml^−1^ (IQR 1.28 to 38.19), and median CRP was 124.1 mg l^−1^ (IQR 60.5 to 203.0). In the overall cohort, both biomarkers increased between day 0 and day 2. Among patients with paired samples, median PCT rose from 5.81 ng ml^−1^ (IQR 1.28 to 38.19) to 6.35 ng ml^−1^ (IQR 1.52 to 30.81; *P*=0.026), while CRP increased from 125.0 mg l^−1^ (IQR 58.1 to 203.6) to 160.0 mg l^−1^ (IQR 97.0 to 226.5; *P*=0.011).

By day 5, both biomarkers declined significantly from baseline. In patients with paired measurements, median PCT decreased from 4.06 ng ml^−1^ (IQR 0.89 to 35.36) to 1.26 ng ml^−1^ (IQR 0.60 to 4.58; *P*<0.001), and CRP decreased from 124.0 mg l^−1^ (IQR 49.3 to 189.8) to 54.5 mg l^−1^ (IQR 33.8 to 88.0; *P*<0.001).

At later timepoints (day 7 and day 10), biomarker levels continued to decline; however, analyses were not extended beyond day 5 due to reduced sample availability and potential bias introduced by differential follow-up.

**Fig. 1.**
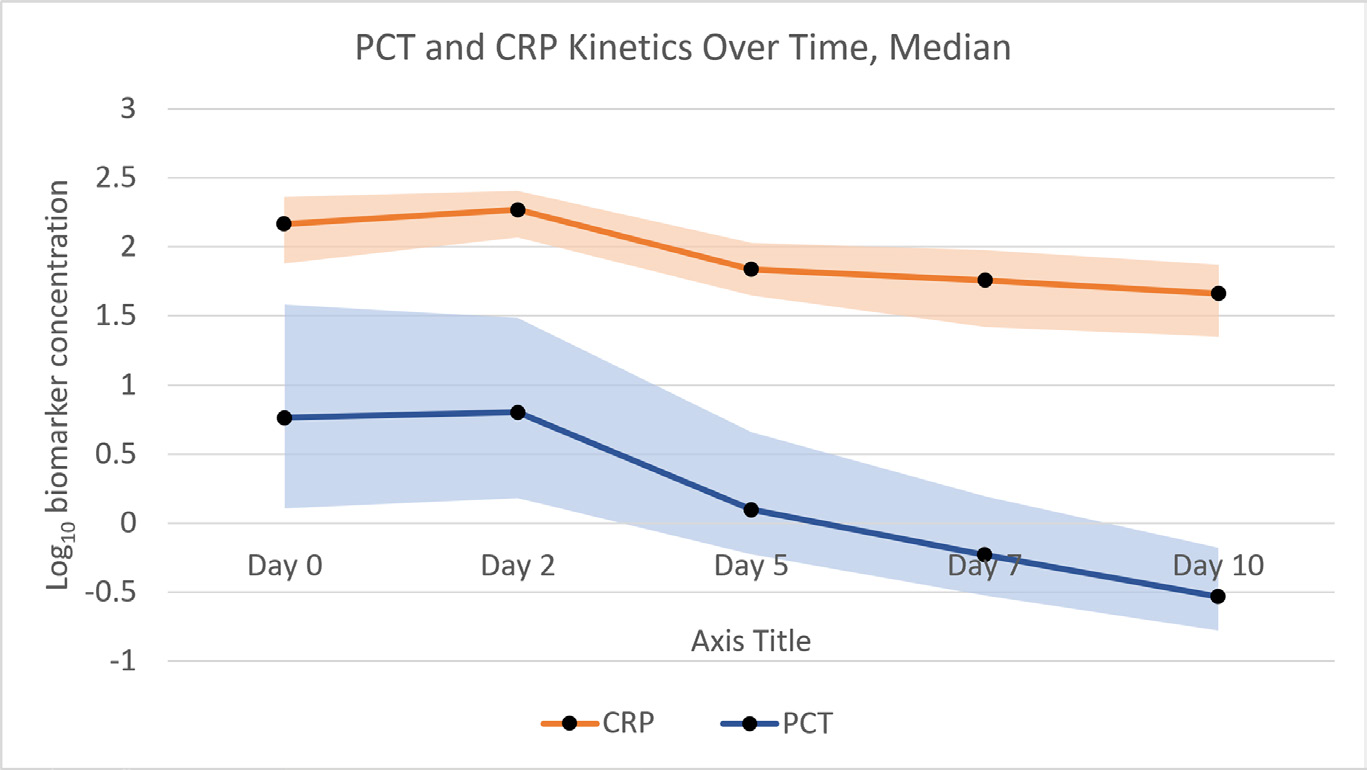
Temporal kinetics of PCT and CRP in Gram-negative bloodstream infection. Lines represent median biomarker concentrations and shaded areas indicate interquartile ranges.

Overall kinetics are visualised in Fig. 1

### Relationship between biomarker kinetics and empiric antimicrobial therapy

Absolute PCT concentrations were lower in patients receiving inappropriate empiric antimicrobial therapy at the time of blood culture positivity than in those receiving active treatment. Median day 0 PCT was 1.76 ng ml^−1^ (IQR 0.66 to 6.11) in the inappropriate therapy group compared with 7.71 ng ml^−1^ (IQR 1.41 to 44.61) in the appropriate therapy group (*P*=0.016). This difference persisted at day 2 [1.53 ng ml^−1^ (IQR 0.85 to 6.21) vs. 8.14 ng ml^−1^ (IQR 1.77 to 35.10); *P*=0.006] and day 5 [0.67 ng ml^−1^ (IQR 0.30 to 1.24) vs. 1.49 ng ml^−1^ (IQR 0.61 to 5.34); *P*=0.008].

CRP did not differ according to empiric antimicrobial appropriateness at any timepoint (day 0, *P*=0.585; day 2, *P*=0.430; day 5, *P*=0.879).

When analysed as a change from baseline, neither biomarker discriminated between appropriate and inappropriate therapy. The median proportional reduction in PCT from day 0 to day 5 was 55.8% (IQR 26.0 to 87.1) in the inappropriate therapy group and 71.1% (IQR −20.2 to 91.7) in the appropriate therapy group (*P*=0.979). Corresponding CRP reductions were 30.6% (IQR 19.4 to 63.6) and 51.8% (IQR 5.0 to 73.5), respectively (*P*=0.219). Absolute change from day 0 to day 5 was also not significantly different for PCT (*P*=0.358) or CRP (*P*=0.153).

### Relationship between biomarker kinetics and source control

There was no significant difference in baseline PCT or CRP according to source control status. Median baseline PCT was 8.32 ng ml^−1^ (IQR 1.21 to 73.86) in patients with a source control concern and 4.27 ng ml^−1^ (IQR 1.34 to 31.79) in those without (*P*=0.208). Median baseline CRP was 119.0 mg l^−1^ (IQR 71.0 to 242.0) and 124.6 mg l^−1^ (IQR 55.4 to 201.0), respectively (*P*=0.698). No differences were observed at day 2 or day 5.

Proportional change analyses similarly showed no association. Median day 5% reduction in PCT was 85.0% (IQR −23.2 to 94.4) in those with a source control concern and 66.3% (IQR −10.9 to 89.7) in those without (*P*=0.225). Corresponding CRP reductions were 38.6% (IQR 3.6 to 68.0) and 50.9% (IQR 5.3 to 73.4), respectively (*P*=0.731). Absolute changes were not significantly different for PCT (*P*=0.069) or CRP (*P*=0.893).

### Exploratory analysis by organism

When grouped as *E. coli* vs. non-*E. coli*, neither baseline biomarker values nor day 5 proportional change differed significantly. Median baseline PCT was 5.89 ng ml^−1^ (IQR 1.38 to 31.79) in *E. coli* bacteraemia and 2.82 ng ml^−1^ (IQR 1.07 to 48.36) in non-*E. coli* bacteraemia (*P*=0.960). Median baseline CRP was 119.8 mg l^−1^ (IQR 60.6 to 201.5) and 125.0 mg l^−1^ (IQR 56.5 to 203.3), respectively (*P*=0.934).

Median day 5 proportional reduction in PCT was 62.3% (IQR −23.4 to 90.8) in *E. coli* infection and 75.5% (IQR −9.0 to 92.2) in non-*E. coli* infection (*P*=0.453). Corresponding CRP reductions were 52.6% (IQR 14.7 to 73.6) and 38.5% (IQR 0.2 to 65.6), respectively (*P*=0.354).

### Exploratory analysis by source of BSI

Baseline PCT did not differ according to source of infection (*P*=0.743). Median baseline PCT was 8.29 ng ml^−1^ (IQR 1.41 to 37.12) in urinary infection, 3.84 ng ml^−1^ (IQR 1.07 to 39.04) in intra-abdominal infection and 2.82 ng ml^−1^ (IQR 0.75 to 32.69) in other sources.

Baseline CRP differed significantly by source (*P*=0.015), with higher values in urinary infection [146.0 mg l^−1^ (IQR 99.0 to 234.5)].

Despite these differences in absolute values, PCT proportional decline from day 0 to day 5 did not differ by source (*P*=0.783). In contrast, CRP proportional decline differed significantly (*P*=0.003), with greater reductions observed in urinary infection.

### Biomarkers, illness severity and outcomes

Baseline PCT was higher in patients admitted to the ICU [26.94 ng ml^−1^ (IQR 1.95 to 63.90)] compared with those not admitted [3.39 ng ml^−1^ (IQR 1.16 to 26.84); *P*=0.014]. This remained independently associated with ICU admission after adjustment for age and sex (adjusted OR 1.24, 95% CI 1.03 to 1.49; *P*=0.024). CRP was not independently associated with ICU admission.

Baseline PCT was also higher in patients with qSOFA ≥2 [14.50 ng ml^−1^ (IQR 2.26 to 93.50)] compared with qSOFA <2 [3.67 ng ml^−1^ (IQR 1.17 to 31.79); *P*=0.017] and remained independently associated after adjustment (adjusted OR 1.21, 95% CI 1.00 to 1.45; *P*=0.046). CRP was not associated with qSOFA.

Baseline PCT was not associated with 30-day mortality (*P*=0.677; adjusted OR 1.05, 95% CI 0.84 to 1.31; *P*=0.668), and CRP was similarly non-predictive. Neither inappropriate empiric therapy nor source control concern was significantly associated with 30-day mortality or readmission.

PCT and CRP proportional change across clinical variables is summarized in Table 2.

**Table 2.** Procalcitonin (PCT) and C-reactive protein (CRP): baseline values and day 5 proportional change across clinical variables

Variable	Group	Baseline PCT (median, IQR)	Baseline CRP (median, IQR)	D5 PCT % change (median, IQR)	D5 CRP % change (median, IQR)	% Change *P*-value (PCT)	% Change *P*-value (CRP)
**Overall cohort**	–	5.81 (1.28–38.19)	124.1 (60.5–203.0)	73.4 (−9.6–91.5)	44.3 (3.7–67.6)	–	–
**Inappropriate empiric therapy**	No	7.71 (1.41–44.61)	124.6 (67.0–205.5)	71.1 (−20.2–91.7)	51.8 (5.0–73.5)	0.979	0.219
	Yes	1.76 (0.66–6.11)	124.0 (49.0–189.0)	55.8 (26.0–87.1)	30.6 (19.4–63.6)	–	–
**Source control concern**	No	4.27 (1.34–31.79)	124.6 (55.4–201.0)	66.3 (−10.9–89.7)	50.9 (5.3–73.4)	0.225	0.731
	Yes	8.32 (1.21–73.86)	119.0 (71.0–242.0)	85.0 (−23.2–94.4)	38.6 (3.6–68.0)	–	–
**Organism**	*E. coli*	5.89 (1.38–31.79)	119.8 (60.6–201.5)	62.3 (−23.4–90.8)	52.6 (14.7–73.6)	0.453	0.354
	Non*-E. coli*	2.82 (1.07–48.36)	125.0 (56.5–203.3)	75.5 (−9.0–92.2)	38.5 (0.2–65.6)	–	–
**Source of infection**	Urinary	8.29 (1.41–37.12)	146.0 (99.0–234.5)	81.4 (12.2–92.8)	49.6 (12.1–71.3)	0.783	0.003
	Intra-abdominal	3.84 (1.07–39.04)	86.0 (47.0–171.5)	58.2 (−15.4–85.6)	36.8 (−5.2–61.4)	–	–
	Other	2.82 (0.75–32.69)	124.0 (55.0–200.0)	69.9 (−10.1–90.2)	43.1 (5.0–66.8)	–	–
**ICU admission**	No	3.39 (1.16–26.84)	120.0 (55.0–200.0)	74.2 (−28.1–91.4)	50.9 (10.0–72.3)	0.014	0.148
	Yes	26.94 (1.95–63.90)	135.0 (70.0–220.0)	61.8 (7.5–88.3)	43.3 (−4.3–72.3)	–	–
**qSOFA ≥2**	No	3.67 (1.17–31.79)	120.0 (55.0–200.0)	75.0 (−9.0–91.4)	50.6 (22.4–70.1)	0.017	0.522
	Yes	14.50 (2.26–93.50)	130.0 (65.0–210.0)	61.8 (−32.9–89.7)	45.6 (−23.3–73.5)	–	–
**30-day mortality**	No	5.74 (1.38–36.14)	120.3 (52.6–193.8)	76.25 (−5.3–91.99)	45.53 (4.53–67.07)	0.677	0.475
	Yes	7.92 (0.68–44.00)	162.0 (93.0–265.0)	47.44 (−16.38–62.69)	27.09 (−13.12–69.07)	–	–

## Discussion

In this cohort of 170 patients with *Enterobacterales* BSIs, early PCT kinetics did not exhibit patterns that could meaningfully influence early clinical management. Although absolute PCT concentrations were higher in patients receiving appropriate empiric therapy at baseline and early follow-up (day 0 median 7.71 vs. 1.76 ng ml^−1^, *P*=0.016), this difference did not translate into consistent differences in biomarker kinetics. PCT followed a broadly similar temporal pattern across the cohort, with a rise between day 0 and day 2 followed by a significant decline by day 5 (*P*<0.001), and neither proportional nor absolute changes over time were associated with antimicrobial appropriateness, source control status, infecting organism or outcome. In contrast, baseline PCT was associated with markers of illness severity, including ICU admission and higher qSOFA scores, but was not predictive of mortality. CRP demonstrated similar temporal behaviour but did not provide additional discriminatory value.

These findings suggest that, in the context of Gram-negative BSIs, early PCT dynamics primarily reflect the host inflammatory response rather than the adequacy of antimicrobial therapy. The observed early rise followed by decline is consistent with known PCT kinetics in systemic infection, where concentrations increase in response to inflammatory cytokine signalling and subsequently fall with source control and immune resolution [28].

The lack of association between PCT kinetics and antimicrobial appropriateness may also reflect the clinical context in which these infections were managed. In this cohort, positive blood cultures were communicated promptly, allowing early microbiology input and rapid adjustment of antimicrobial therapy. As a result, the duration of truly inappropriate therapy was likely short, limiting the opportunity for biomarker trajectories to diverge. This is an important consideration, as the potential utility of PCT as a marker of treatment adequacy may be attenuated in settings with robust diagnostic stewardship and early specialist involvement [9,29].

Our findings differ from studies in other infectious syndromes, particularly lower respiratory tract infections, where PCT kinetics have been associated with clinical response and used to guide antimicrobial discontinuation [30,31]. In patients with nosocomial pneumonia, greater early declines in PCT have been linked to treatment success, and thresholds for percentage reduction have been proposed as predictors of clinical efficacy [32]. Similarly, in critically ill cohorts, persistently elevated or non-declining PCT has been associated with adverse outcomes [33,34]. The absence of a comparable association in BSIs may reflect differences in pathophysiology, including the rapid systemic dissemination of infection, heterogeneity in source and variability in host response.

Importantly, while PCT kinetics did not inform treatment adequacy, baseline concentrations were associated with illness severity. Higher initial PCT levels were observed in patients requiring ICU admission and in those with higher qSOFA scores, consistent with previous work demonstrating a relationship between PCT and sepsis severity [34,35]. This supports a role for PCT as a marker of physiological derangement at presentation rather than as a dynamic indicator of treatment response. However, baseline PCT was not independently associated with mortality in this cohort, suggesting that its prognostic value may be limited when considered in isolation [35].

We also observed that biomarker behaviour was largely consistent across infecting organisms and source of infection, except for higher CRP values in urinary-source bacteraemia and greater CRP decline over time in this group. These findings likely reflect differences in inflammatory burden and source-specific host response rather than meaningful differences in treatment dynamics. Notably, PCT kinetics remained similar across sources, further reinforcing the lack of discriminatory utility for early management decisions.

Several limitations should be acknowledged. First, although the cohort included 170 patients, the availability of complete serial biomarker measurements declined at later timepoints, resulting in smaller paired datasets for day 5 and subsequent analyses. This limits statistical power to detect differences in biomarker kinetics and raises the possibility that clinically relevant divergence may have been missed. Second, the cohort was heterogeneous with respect to source of infection, organism and illness severity, which, while reflective of real-world practice, introduces biological variability that may obscure more subtle associations between biomarker behaviour and treatment response. Third, we did not adjust for important host factors known to influence PCT concentrations, including renal impairment, systemic inflammatory conditions, recent surgery or critical illness unrelated to infection, all of which may have contributed to the wide inter-individual variability observed [35]. Fourth, source control was assessed as a binary variable at baseline, and the timing of its resolution was not captured. This may have influenced biomarker trajectories independently of antimicrobial therapy. In addition, although proportional change was examined, the use of percentage reduction may be sensitive to baseline values, particularly in patients with low initial PCT levels.

From a clinical perspective, these findings suggest that early serial PCT measurement is unlikely to provide actionable information to guide initial antimicrobial decision-making in *Enterobacterales* BSIs. In settings with timely microbiological diagnostics and early specialist input, reliance on PCT kinetics to infer treatment adequacy appears unwarranted. Rather than reflecting therapeutic response, early PCT dynamics may reflect the host’s inflammatory response to established infection. Future studies should prioritize larger cohorts with more complete longitudinal sampling and integrate biomarker data with clinical, microbiological and host-response parameters. Approaches that combine biomarker kinetics with measures of pathogen burden or genomic resistance may offer greater potential to support precision antimicrobial therapy.

## Abbreviations

AST: antimicrobial susceptibility testing
BSIs: bloodstream infections
CRP: C-reactive protein
ICU: intensive care unit
IQR: interquartile range
MALDI-TOF: matrix-assisted laser desorption ionisation time-of-flight
PCT: procalcitonin
qSOFA: quick Sequential Organ Failure Assessment
